# Negative association of hemoglobin levels with mortality in older Japanese patients with dysphagia: a retrospective cohort study

**DOI:** 10.3389/fmed.2026.1742752

**Published:** 2026-03-05

**Authors:** Ya-Ting Yuan, Miao Wang, Xian-Zhi Liu, Xiao Cheng, Xue-Ying Cai, Han-Han Chen, Xiao-Bin Zhang

**Affiliations:** 1 Department of Pulmonary and Critical Care Medicine, Zhongshan Hospital Xiamen University, School of Medicine, Xiamen University, Xiamen, Fujian, China; 2 School of Medicine, Xiamen University, Xiamen, Fujian, China; 3 Xiang'an Hospital of Xiamen University, School of Medicine, Xiamen University, Xiamen, Fujian, China; 4 The School of Clinical Medicine, Fujian Medical University, Fuzhou, Fujian, China

**Keywords:** dysphagia, hemoglobin, mortality, older adults, retrospective cohort study

## Abstract

**Objective:**

This secondary analysis aimed to examine the association between hemoglobin levels and mortality in older Japanese individuals with dysphagia.

**Methods:**

Data from 253 patients with clinically confirmed dysphagia between January 2014 and January 2017 were analyzed. Participants were divided into three tertiles based on their hemoglobin levels (T1: 5.4–10.2 g/dL; T2: 10.2–12.0 g/dL; T3: ≥12.0 g/dL). Cox proportional hazards regression was used to evaluate mortality risk, supplemented by Kaplan–Meier survival analysis and subgroup interaction assessments.

**Results:**

The study included 154 female and 99 male patients, with a median age of 83 years. The cohort demonstrated a significant inverse relationship between hemoglobin levels and mortality. After full covariate adjustment, each 1 g/dL increase in hemoglobin level corresponded to a 14% mortality risk reduction (HR = 0.86, 95% CI: 0.77–0.97, *P* = 0.013). Compared to T1, adjusted hazard ratios for T2 and T3 were 0.53 (95% CI: 0.34–0.84, *P* = 0.007) and 0.48 (95% CI: 0.27–0.83, *P* = 0.009), respectively (*P* for trend = 0.005). Median survival differed significantly across tertiles: 212 days (T1), 540 days (T2), and >1463 days (T3) (*P* < 0.0001). Subgroup analyses confirmed the robustness without significant interactions.

**Conclusion:**

Hemoglobin concentration was independently and inversely associated with mortality in older Japanese patients with dysphagia. These findings underscore the prognostic value of hemoglobin in this population.

## 1 Introduction

Dysphagia, an impairment involving challenges or unease during swallowing, poses the risk of accidental passage of ingested materials or secretions into the respiratory tract. Approximately 8% of individuals worldwide experience dysphagia, and nearly one million new cases (1 out of 25 adults) are diagnosed each year in the US. In community-dwelling older individuals, the prevalence is approximately 15%, whereas it increases to approximately 30% among patients who are hospitalized (1). Dysphagia impairs the quality of life, nutritional status, and overall health, significantly contributing to morbidity and mortality. The healthcare costs and hospitalization rates associated with dysphagia and its complications are substantial (2, 3). Many older adults with progressive neurological diseases have significant but often unrecognized dysphagia, which increases the risk of aspiration pneumonia and malnutrition (4).

Hemoglobin is essential for oxygen delivery and tissue perfusion, and is crucial for the management of patients who are critically ill (5). Several studies have documented the association of anemia with adverse clinical consequences across various disorders, including chronic heart failure (CHF) (6), acute myocardial infarction (AMI) (7), and chronic kidney disease (CKD) (8). Hemoglobin supports innate immunity by sequestering iron to limit bacterial growth, maintaining hemodynamic stability, and facilitating antibiotic delivery, and therefore, maintaining optimal hemoglobin levels is essential for patients who are critically ill (5). Furthermore, anemia could be linked to multiple functional declines, including poorer mobility, weaker muscles, elevated fall propensity, hindered rehabilitation progress, diminished self-assessed physical capacity, and overall cognitive deterioration in older individuals who are non-critically ill (9).

Although hemoglobin levels are correlated with multiple disease states, their association with mortality in older patients with dysphagia has not been studied adequately. Using propensity score matching, this cohort analysis revealed that percutaneous endoscopic gastrostomy (PEG) is associated with markedly prolonged survival among older adults experiencing swallowing difficulties (10). We performed a secondary cohort analysis to evaluate hemoglobin-mortality associations among Japanese patients.

## 2 Materials and methods

### 2.1 Data source

Data were collected from the Dryad Digital Repository, an open-access platform that facilitates data acquisition, according to its terms of service. The specific data package “Comparison of long-term outcomes between enteral nutrition via gastrostomy and total parenteral nutrition in older persons with dysphagia: A propensity-matched cohort study” (available at https://doi.org/10.5061/dryad.gg407h1) was accessed for this study. The findings of this study were published in 2019 (10).

### 2.2 Study design and participants

This retrospective single-center cohort study included patients aged ≥ 65 years with severe dysphagia who underwent PEG or total parenteral nutrition (TPN) administration between January 2014 and 2017. Dysphagia diagnosis required clinical assessment by physicians, nurses, speech-language pathologists, and video-fluoroscopic confirmation. Dysphagia diagnosis relied on pre-existing classifications within the source dataset, which did not document video-fluoroscopic examination protocols. The exclusion criteria included terminal malignancy, pre-2014 PEG placement, and gastrostomy for decompression. The Miyanomori Memorial Hospital Ethics Committee approved the anonymized data analysis study (informed consent was waived).

### 2.3 Procedures

The choice between PEG feeding and TPN administration was based on thorough discussions between patients (or their families) and clinicians. Clinical assessments by the medical team guided the decisions on the delivery of nutritional therapy. Patient data, including age, sex, height, weight, comorbidities, and laboratory findings, were obtained from medical records.

Hemoglobin levels were measured using blood tests conducted within seven days preceding the initiation of either PEG feeding or TPN administration. The principal aim was to evaluate the mortality after procedure initiation throughout a pre-specified follow-up period. Participants were divided into three tertiles based on their enrollment hemoglobin levels: T1 (5.4–10.2 g/dl), T2 (10.2–12.0 g/dl), and T3 (≥12.0 g/dl).

### 2.4 Statistical analysis

Secondary analyses were performed using publicly available datasets. Categorical data -were summarized using percentages, while continuous measures were presented as mean ± standard deviation (SD) or median (inter-quartile range; IQR). Continuous variables across the three tertiles were analyzed using one-way ANOVA. Categorical measures were analyzed using chi-square tests for baseline characteristic comparisons. The association between hemoglobin levels and mortality risk in patients with dysphagia was assessed using Cox proportional hazards regression modeling. Kaplan-Meier curves with log-rank tests were used to visualize and compare survival distributions. In multivariate analyses, confounding factors were an important issue and different statistical models were used to verify the stability of the results. The covariates in the regression models were corrected according to three criteria: variables with *P*-values less than 0.1 in univariate analyses, variables whose matched odds ratios would change by at least 10% after inclusion in the model, and variables selected based on clinical constraints. Subgroup interactions were examined using likelihood ratio tests. Statistical analyses were performed using the R software (unspecified version; R Foundation for Statistical Computing, Vienna, Austria) and Free Statistics v2.1. A two-sided α level of 0.05 defined statistical significance, with all cited p-values falling below this cutoff.

## 3 Results

### 3.1 Study participants and baseline characteristics

Table 1 summarizes the baseline characteristics of the study cohort, which comprised 253 patients (99 males, 154 females). The mean patient age was 83.1 ± 9.3 years. Among the censored patients, the median follow-up period was 601 days (404–823), whereas the median survival duration was 306 days (116–609). Significant differences (*P* < 0.05) were observed among the tertiles regarding age, ischemic heart disease (IHD), chronic heart failure, chronic kidney disease, C-reactive protein (CRP) levels, total cholesterol (Tc), serum albumin (Alb), and total lymphocyte count (TLC).

**Table 1:** Baseline characteristics of patients.

**Variables**	**Total (*n* = 253)**	**Hb, T1 (5.4 ~10.2) g/dl (*n* = 82)**	**Hb, T2 (10.2 ~12) g/dl (*n* = 84)**	**Hb, T3(≥12) g/dl (*n* = 87)**	***P***
Age (year)	83.1 ± 9.3	84.7 ± 6.8	84.6 ± 7.9	80.0 ± 11.7	<0.001
**Sex**, ***n*** **(*****%*****)**					
Male	99 (39.1)	32 (39)	29 (34.5)	38 (43.7)	0.471
Female	154 (60.9)	50 (61)	55 (65.5)	49 (56.3)	
CI, *n* (%)	133 (52.6)	41 (50)	40 (47.6)	52 (59.8)	0.24
Dement, n (%)	102 (40.3)	38 (46.3)	37 (44)	27 (31)	0.089
Asp, *n* (%)	94 (37.2)	33 (40.2)	33 (39.3)	28 (32.2)	0.492
IHD, *n* (%)	47 (18.6)	23 (28)	14 (16.7)	10 (11.5)	0.019
CHF, *n* (%)	107 (42.3)	45 (54.9)	38 (45.2)	24 (27.6)	0.001
Lung, *n* (%)	19 (7.5)	7 (8.5)	5 (6)	7 (8)	0.797
Liver, *n* (%)	15 (5.9)	6 (7.3)	4 (4.8)	5 (5.7)	0.779
CKD, *n* (%)	53 (20.9)	36 (43.9)	13 (15.5)	4 (4.6)	<0.001
Alb (g/dl)	3.1 ± 0.6	2.7 ± 0.5	3.1 ± 0.5	3.5 ± 0.6	<0.001
CRP (mg/dl)	1.0 (0.3, 3.3)	2.0 (0.5, 5.0)	1.2 (0.4, 3.7)	0.4 (0.1, 1.5)	<0.001
BMI	19.2 ± 3.3	19.2 ± 3.4	19.0 ± 3.3	19.4 ± 3.3	0.719
TC (mg/dl)	156.0 ± 39.2	138.1 ± 35.8	156.5 ± 40.5	172.4 ± 33.9	<0.001
TLC (mm^3^)	1333.1 ± 703.7	1194.2 ± 809.7	1286.8 ± 644.2	1508.8 ± 617.5	0.011
Survival (day)	306.0 (116.0, 609.0)	194.5 (53.0, 414.8)	343.0 (136.5, 598.8)	405.0 (182.5, 715.0)	<0.001
**Status**, ***n*** **(*****%*****)**					
Death	115 (45.5)	19 (23.2)	38 (45.2)	58 (66.7)	<0.001
Alive	138 (54.5)	63 (76.8)	46 (54.8)	29 (33.3)	

CI, cerebrovascular disease; Dement, severe dementia; Asp, aspiration pneumonia; IHD, ischemic heart disease; CHF, chronic heart failure; lung, chronic lung disease; liver, chronic liver disease; CKD, chronic kidney disease; Alb, serum albumin; TLC, total lymphocyte count; TC, total cholesterol; Hb, hemoglobin; CRP, C-reactive protein; BMI, body mass index; survival, survival time.

### 3.2 Kaplan–Meier curve

**Figure 1:**
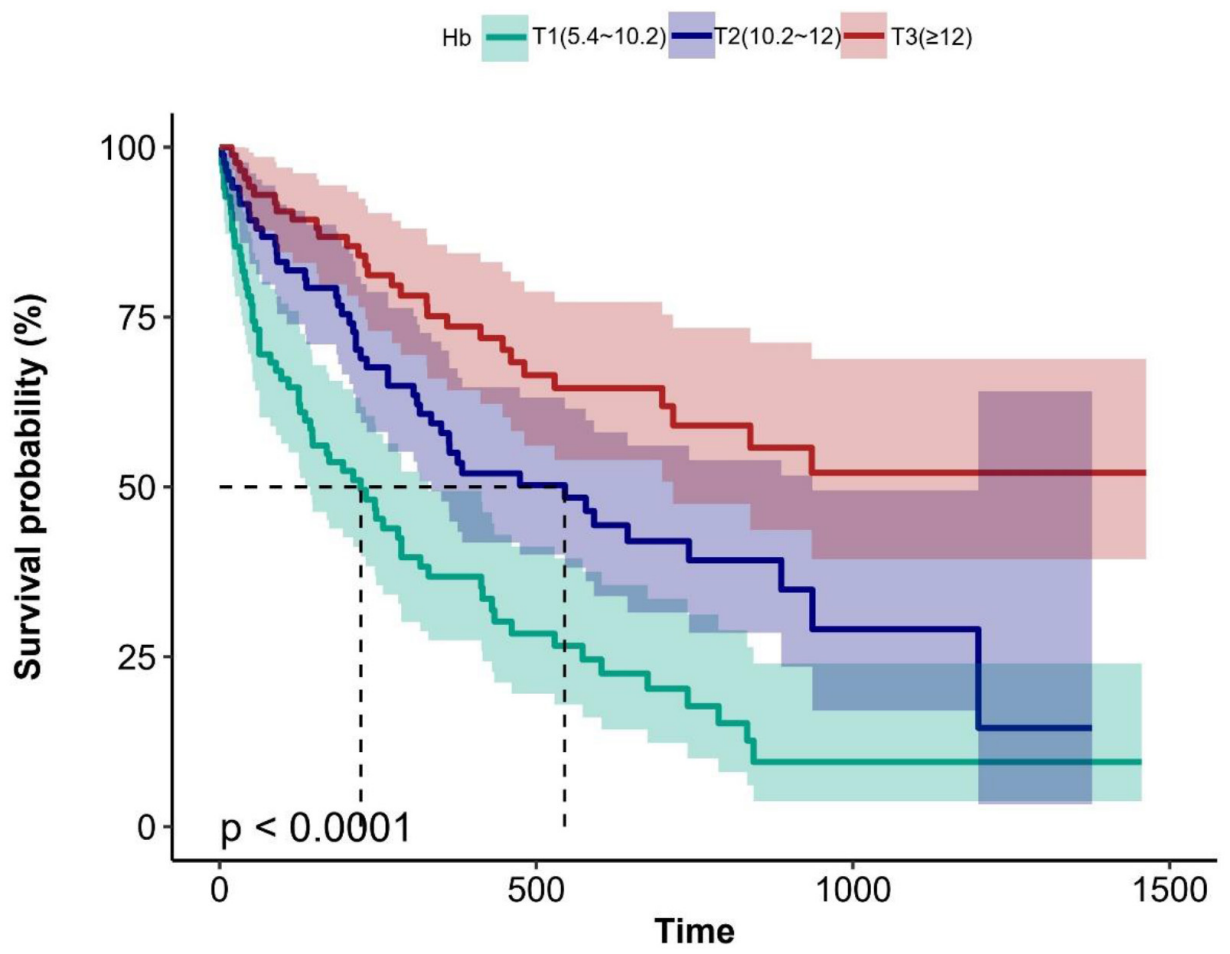
Kaplan–Meier survival analysis for mortality with Hb in three groups. Hb, Hemoglobin.

The Kaplan–Meier curve in Figure 1 shows that the T1 group demonstrated a significantly shorter median survival than the T2 and T3 groups. The median survival durations for T1, T2, and T3 groups were 212, 540, and >1463 days, respectively (*P* < 0.0001).

### 3.3 Association between hemoglobin and mortality in various models

Table 2 shows the hazard ratios (HR) and 95% confidence intervals (95% Cl) associated with the risk of mortality in patients with dysphagia based on hemoglobin levels. Four models were used for hemoglobin as continuous and categorical variables. No variables were chosen for adjustment in Model I. Age, sex, and body mass index (BMI) were chosen for adjustment in Model II. Demographic variables (age, sex, and BMI) and medical history (CHF, IHD, lung, liver, CKD, cerebrovascular diseases, severe dementia, and aspiration pneumonia) were adjusted for in Model III. All variables included (age, sex, BMI, CRP, Alb, TC, and TLC) were adjusted for in model IV. Table 2 presents the results of the analytical models. Univariable Cox regression revealed an inverse relationship between hemoglobin elevation and mortality risk (HR 0.77, 95% CI 0.71–0.84; *P* < 0.001). After full covariate adjustment in the multivariable Cox model, a significant protective association was observed (HR 0.86, 95% CI 0.77–0.97; *P* = 0.013). When compared with the lowest hemoglobin group T1 (5.4–10.2), the adjusted HR values for hemoglobin and mortality in the T2 (10.2–12.0) and T3 (≥ 12.0) groups were 0.53 (95% CI: 0.34–0.84, *P* = 0.007) and 0.48 (95% CI: 0.27–0.83, *P* = 0.009, *P* for trend = 0.005), respectively.

**Table 2:** Association between Hb and mortality in different models.

**Variable**	Model I		Model II		Model III		Model IV	
	**HR (95% CI)**	***P***	**HR (95% CI)**	***P***	**HR (95% CI)**	***P***	**HR(95% CI)**	***P***
Hb	0.77 (0.71 ~ 0.84)	<0.001	0.79 (0.73 ~ 0.86)	<0.001	0.8 (0.73 ~ 0.89)	<0.001	0.86 (0.77 ~ 0.97)	0.013
Hb (T1, 5.4~10.2)	1 (Ref)	–	1 (Ref)	–	1 (Ref)	–	1 (Ref)	–
Hb (T2, 10.2 ~ 12.0)	0.52 (0.35 ~ 0.76)	0.001	0.5 (0.34 ~ 0.74)	<0.001	0.48 (0.33 ~ 0.75)	0.001	0.53 (0.34 ~ 0.84)	0.007
Hb(T3, ≥12.0)	0.27 (0.18 ~ 0.43)	<0.001	0.31 (0.2 ~ 0.49)	<0.001	0.36 (0.46 ~ 0.76)	<0.001	0.48 (0.27 ~ 0.83)	0.009
*P* for trend	–	<0.001	–	<0.001	–	<0.001	–	0.005

Model I: non-adjusted.

Model II: adjusted for age, sex and BMI.

Model III: adjusted for model II plus CI, CHF, IHD, Asp, lung, liver, CKD, and dementia.

Model IV: adjusted for model III plus CRP, Alb, TC, and TLC.

### 3.4 Subgroup analyses

**Figure 2:**
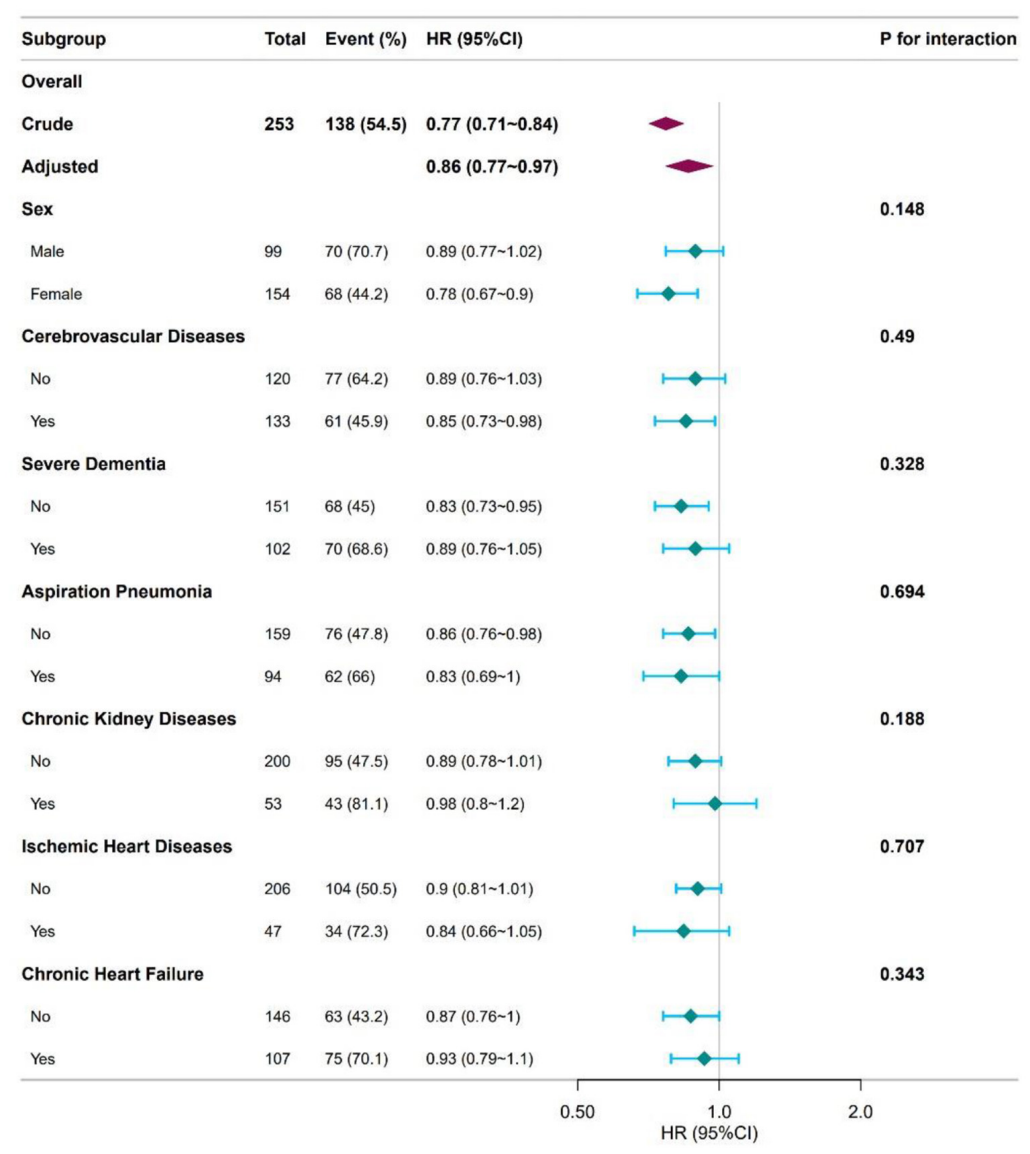
Subgroup analyses of hemoglobin associated with mortality. Hazard ratios (HRs) were adjusted for sex, cerebrovascular diseases, severe dementia, aspiration pneumonia, chronic kidney diseases, ischemic heart disease and chronic heart failure.

To evaluate the robustness of the association between hemoglobin level and dysphagia mortality (Figure 2), subgroup and interaction analyses were conducted. The covariates adjusted for in these analyses included sex, cerebrovascular disease, severe dementia, aspiration pneumonia, CKD, IHD, and CHF. Categorical stratification variables were excluded from corresponding subgroup analyses. No significant interactions were detected among the seven subgroups examined. This finding shows consistent results, and is illustrated in Figure 2.

## 4 Discussion

Hemoglobin operates at the critical interface of molecular biology and human longevity, serving not merely as an oxygen carrier but as a fundamental regulator of systemic physiological resilience. Beyond erythrocytic confinement and laboratory metrics, hemoglobin interfaces with aging via dysregulated erythropoiesis, xidative stress amplification, vascular homeostasis perturbation, and impaired tissue oxygenation. Declining hemoglobin efficacy and erythrocyte integrity in aging serve as biomarkers for multisystem physiological decline, contributing mechanistically to frailty phenotypes, organ functional reserve depletion, and advancement of age-related comorbidities (5, 11).

Our investigation identified a significant inverse relationship between hemoglobin concentration and mortality risk. Univariate analysis of continuous hemoglobin levels demonstrated a significant 23% mortality risk reduction per unit increase in patients with dysphagia (HR 0.77, 95% CI 0.71–0.84, *P* < 0.001). The inverse correlation persisted significantly post full adjustment (multivariate HR 0.86, 95% CI 0.77-0.97, *P* = 0.013), conferring 14% mortality risk reduction. Similarly, when hemoglobin was categorically analyzed, the unadjusted model revealed a significant inverse relationship with dysphagia-related mortality. Compared with the T1 group, the T3 group exhibited a 73% reduction in mortality risk. Hemoglobin levels were consistently linked to better mortality protection in patients with dysphagia after full multivariate adjustment, with the inverse correlation remaining robust. Specifically, the T2 and T3 groups demonstrated 47% and 52% lower risks of all-cause mortality than the T1 group, respectively. Consistently, the T1 group had a significantly shorter median survival than the T2 and T3 groups. Subsequent interaction analyses across the seven predefined subgroups (sex, cerebrovascular disease, severe dementia, aspiration pneumonia, CKD, IHD, and CHF) revealed no significant effect modifications.

Anemia is highly prevalent among the older population, with estimates ranging from 2.9 to 60.1% of older adults affected, depending on the specific population examined (12, 13). Our findings indicate that nearly two-thirds of older individuals with dysphagia have hemoglobin concentrations of < 12 g/dL. Key anemia origins in this cohort involve micronutrient deficits (iron, cyanocobalamin, and folate), reduced renal function, ACI, and airborne pollutants (particulate matter and NO_2_) (12). Furthermore, approximately 33% of anemia cases among older adults in the US defy the etiological classification despite thorough investigation, with no definitive etiology established. The cohort in our investigation comprised older patients with dysphagia, and the elevated incidence of anemia observed in this group may be predominantly due to inadequate nutritional intake. Furthermore, multiple studies have documented associations between anemia and various adverse outcomes, including disability (14–16), mobility impairment (17, 18), cognitive decline (19–22), diminished quality of life (22), and increased mortality (18, 23).

Our study identified an inverse association between hemoglobin concentration and mortality risk in older patients with dysphagia. This finding aligns with those of several previous studies in this domain (24, 25). However, contrasting results were reported by Sheng et al. (5), where they observed a nonlinear (U-shaped) relationship between hemoglobin levels and in-hospital mortality in a cohort with general sepsis. Specifically, their analysis reveals an inflection point at 10.2 g/dl. Below this threshold, each 1 g/dL decrease in hemoglobin level corresponded to a 4.6% increase in the in-hospital mortality risk. However, above this value, each 1 g/dL increment was linked to a 7.6% increase in mortality risk. The discrepancy between our findings and those of Sheng et al. (5) may be attributed to two primary factors: (1) distinct study populations where our cohort comprised older adults with dysphagia, whereas Sheng et al. focused on patients who are critically ill with sepsis, and (2) limited statistical power, where our study may have been constrained by an insufficient sample size for detecting complex relationships.

This cohort investigation documented the protective effect of hemoglobin against mortality in older patients with dysphagia, with a significantly reduced mortality risk at higher levels. Notably, reduced hemoglobin levels demonstrated an independent association with elevated mortality after adjustment for covariates such as age, coexisting medical conditions, and laboratory parameters. This study provides novel insights into the prognostic role of hemoglobin in dysphagia-related outcomes, highlighting its potential as a modifiable risk factor in this vulnerable population. Hemoglobin level assessment is a main component of clinical blood tests, and is affordable for most patients, and it offers clinicians a basis for prognostic evaluation in older individuals with swallowing difficulties and administer such approaches into routine practice to enhance quality of care and patient outcomes.

This study has several limitations. The study concentrated exclusively on older patients with dysphagia which limited the generalizability of our findings, thereby limiting their relevance to wider demographic groups, such as patients with Parkinson's disease. Second, potential selection bias may exist due to relying on a single hemoglobin measurement obtained within the first week of hospitalization, with no subsequent assessments. This precludes the evaluation of how temporal variations in hemoglobin levels may influence outcomes. Furthermore, being a single-center study with a limited sample size necessitates validation through larger multicenter studies. Moreover, as this study was conducted exclusively in an older Japanese patients, the results may not apply to other populations or countries, requiring further validation through additional studies.

## 5 Conclusions

Our findings demonstrate an inverse association between hemoglobin levels and mortality risk in older Japanese patients with dysphagia. Lower hemoglobin concentrations were independently associated with an elevated mortality risk, even after accounting for multiple potential confounders. These results highlight the clinical importance of closely monitoring hemoglobin levels in older adults, particularly in hospitalized individuals, implementing regular assessments, and promptly addressing anemia.

## Data Availability

The original contributions presented in this study are included in this article/supplementary material, further inquiries can be directed to the corresponding author.
